# Socio-demographic influence of food additives acceptance and avoidance among the Lebanese population: A cross-sectional study

**DOI:** 10.1371/journal.pone.0326629

**Published:** 2025-06-30

**Authors:** Reham E. Kotb, Souheil Hallit, Farah Asmar, Melissa Fakhry, Chadia Haddad, Pascale Salameh, Samar Younes, Mohammed Hossni, Mohamad Abdelkhalik, Michelle Cherfane, Katia Iskandar

**Affiliations:** 1 Department of Environmental and Public Health, College of Health Sciences, Abu Dhabi University, Abu Dhabi, UAE; 2 Department of Primary Health Care, High Institute of Public Health, Alexandria University, Alexandria, Egypt; 3 School of Medicine and Medical Sciences, Holy Spirit University of Kaslik, Jounieh, Lebanon; 4 Psychology Department, College of Humanities, Effat University, Jeddah, Saudi Arabia; 5 Applied Sciences Research Center, Applied Science Private University, Amman, Jordan; 6 Department of Nutrition, School of Public Health, Lebanese University, Fanar, Lebanon; 7 Research Department, Psychiatric Hospital of the Cross, Jal Eddib, Lebanon; 8 Gilbert and Rose-Marie Chagoury School of Medicine, Lebanese American University, Byblos, Lebanon; 9 INSPECT-LB (Institut National de Santé Publique, d’Épidémiologie Clinique et de Toxicologie-Liban), Beirut, Lebanon; 10 Department of Primary Care and Population Health, University of Nicosia Medical School, Nicosia, Cyprus; 11 Faculty of Pharmacy, Lebanese University, Hadat, Lebanon; 12 Department of Biomedical Sciences, School of Pharmacy, Lebanese International University, Bekaa, Lebanon; 13 Department of Pharmacy Practice and Clinical Pharmacy, Specialty of Pharmaceutical Management and Economics, Faculty of Pharmacy, Egyptian Russian University, Badr City, Cairo Governorate, Egypt; 14 Department of Pharmaceutical Sciences, School of Pharmacy, Lebanese International University, Beirut, Lebanon; 15 Department of Health and Social Work, School of Public Health, Lebanese University, Fanar, Lebanon; Yale University School of Medicine, UNITED STATES OF AMERICA

## Abstract

Consumers’ exposure to food additives (FAs) through food consumption is becoming inevitable, triggering concerns about their safety. This study aims to evaluate the factors influencing food choices, the level of knowledge among Lebanese consumers about FAs, and the socio-demographic determinants of FAs acceptance and avoidance. A cross-sectional web-based study enrolling 601 participants was conducted online using the snowball technique. The questionnaire consisted of four parts, including the patient socio-demographic characteristics, knowledge about FAs’ definition and label content, the drivers of food choices, and FA acceptance and avoidance. Results showed varying levels of FAs knowledge across different age groups. Consumers indicated insufficient education about FAs (64.9%) and inadequate labeling (59.4%) are primary challenges in understanding food labels. Taste (75%) and price (62.2%) rather than FA content (29.3%) impacted food choices. Multivariate analysis showed that males were more likely to avoid FAs (β = 1.467, p = 0.016), as were university degree holders (β = 1.809, p = 0.012). Dieticians showed lower FA acceptance (β = −1.875, p = 0.031). The study highlights how socio-demographic factors and knowledge influence attitudes toward food adulteration, underscoring the need for targeted education, effective labeling policies, and further research on risk perception and behavioral change.

## 1. Introduction

The growing consumption of processed and ultra-processed food, often characterized by their reliance on food additives (FAs), has become a significant public health concern [1–5]. FAs play a crucial role in shaping modern dietary trends [1–5]. While processed foods may contain minimal additives, ultra-processed foods are typically rich in FAs like artificial flavors, emulsifiers, and preservatives [1,3].

In the United States, FAs account for 57% of daily energy intake in adult US citizens and 67% of youth, according to the National Health and Nutrition Examination Survey (NHANES) [2]. In Europe, consumption ranges between 15% in the adult Romanian population and 57% in the United Kingdom (UK) [2].

FAs are substances added to processed food to preserve and improve their taste, freshness, texture, and appearance [3]. They can be synthetic or derived from natural sources such as plants, animals, and minerals [6,7]. The World Health Organization (WHO) classifies FAs into three broad categories: flavoring agents, enzyme preparations, and other FAs, including colorants, sweeteners, and preservatives [6].

The growing reliance on FAs is influenced by numerous factors, including global population growth [8], dietary shifts [9], changing lifestyles [10], and consumers’ desire for variety and convenience [11]. Urbanization [10], economic conditions [12], and climate changes affecting agriculture [13] have further contributed to the demands for processed and ultra-processed food [9,14], in which FAs are commonly used to enhance shelf life, flavor, and appearance. The widespread use of FAs rich food content necessitates urgent scrutiny for three critical reasons.

First, there are significant health implications. While many FAs are safe within regulated limits, emerging research suggests potential health risks associated with excessive consumption and long-term exposure, particularly in children and chronic consumers [6,7,15–19]. Certain synthetic colorants and preservatives have been linked to hypersensitivity reactions, behavioral changes in children, and possible carcinogenic effects in animal studies associated with chronic exposure [15–19].

Second, regulatory challenges persist globally. To ensure the safety of FA use, international regulatory bodies, such as the Codex Alimentarius Commission, the Joint FAO/WHO Expert Committee on Food Additives (JECFA) [20,21], and the European Food Safety Authority (EFSA) [22] have established guidelines for acceptable levels of use and the Acceptable Daily Intake (ADI) [20]. However, global regulatory disparities create uneven safety standards, with developing nations often lacking enforcement capacity. In Lebanon, LIBNOR (the Lebanese Standards Institution) adopts the Codex Alimentarius and JECFA guidelines, but implementation gaps persist, creating uneven consumer protection [23].

Third, despite these regulatory efforts and continuous safety assessment and monitoring [20–22], consumers’ concerns about the health implications of FAs and behavioral gaps persist [24–34]. Factors such as knowledge about FAs [35–37], education [37,38], gender [38], age [39], sources of information about FAs [33,34], and trust in regulators [24] influence the perception of FAs and the growing trend toward additive-free products [33,34].

In Lebanon, previous research has highlighted low knowledge levels [40] and awareness about nutrition labels, including E-numbers [41]. While strict enforcement of national food safety regulation and consumer protection is lacking, this study aims to evaluate the factors influencing food choices, the level of knowledge among Lebanese consumers about FAs, and the socio-demographic determinants of FAs acceptance and avoidance. Addressing these gaps will provide insights into designing targeted public health campaigns and policies to improve food literacy and support healthier eating behaviors in the Lebanese population.

## 2. Materials and methods

### 2.1. Study design, setting, and participants

A cross-sectional analytical study was conducted from May 1 2023 till July 31 2023. The questionnaire was developed on Google Forms. The participants were Lebanese citizens 18 years and above who were eligible to enroll in the study regardless of their level of education, profession, and gender. Data were collected using the snowball sampling technique [42] by asking university students to share the link of the online survey with their families and social networks using various social media platforms (WhatsApp, Facebook, and LinkedIn).

### 2.2. Sample size calculation

The minimum sample size was calculated using the G-Power software, version 3.0.10. The calculated effect size was 0.0526, expecting squared multiple correlations of 0.05 (R2 deviation from 0) related to the Omnibus test of multiple regression. The minimum necessary sample was n = 400, considering an alpha error of 5%, a power of 80%, and allowing 20 predictors in the model.

### 2.3. Questionnaire

The questionnaire was pilot tested with a small sample (n = 30) of participants representing the target population. The pilot study assessed question clarity, identify ambiguous items, estimate completion time, and evaluate the reliability of measurement scales. Based on pilot feedback, minor wording adjustments were made to improve comprehension while maintaining the original meaning of items.

The final questionnaire was formulated in English based on similar published studies [11,24,28,32,36]. It consisted of four sections: The first section examined the socio-demographic characteristics of participants, such as age, gender, marital status, children or not, holding a university degree, and being a dietician. The second section consisted of seven questions (No/Yes) formulated to evaluate the consumer level of knowledge of FAs’ definition and label content. These included the following correct answers: 1) Food additives are natural or artificial substances added intentionally to food products for technological purposes. 2) Natural food additives are derived from living sources, and artificial additives are manufactured in laboratories. 3) Food additives are added for better taste or flavor. 4) Food additives are added for better color or shape. 5) Food additives are added to extend the shelf-life of processed food. 6) A ‘preservative-free’ label means that the product contains other additives but no preservatives. 7) The label “calorie-free” means the beverage is contains zero calories.

The level of knowledge was classified based on the percentage of correct answers marked as 1 point [30] for poor: < 40%, good: between ≥40% and <60%; very good: between >60 and ≤80 and excellent: > 80%. The third section consisted of two multiple-choice questions to investigate (1) the drivers of food choices among Lebanese consumers, including price, taste, convenience, FA content, nutritional value, and brand, and examine (2) the perceived challenges in understanding food label that included, insufficient labeling, insufficient education about FAs, and difficulties understating FAs. The fourth section consisted of ten questions about FA avoidance and eight about FA acceptance. These questions were formulated according to similar studies [18,26,30]. A 5-point Likert scale used the following options ranging from 1 = Totally disagree; 2 = Disagree; 3 = Neutral; 4 = Agree; and 5 = Totally agree. Acceptance questions were as follows: 1) Without food additives, the variety of food products would be severely limited, 2) All foods contain additives, so there is no way to avoid them, 3) The additive content of foods labeled as “Diet” is very low, 4) Food additives obtained from genetically modified organisms (GMOs) can be used safely in convenience foods, 5) The food label does not help identify the different sources of food additives, 6) Food products labeled as “Halal Certified” assure consumers of the Muslim faith that the ingredients, including additives, meet their religious requirements, 7) The food label “suitable for vegetarians” means that all ingredients and food additives are from vegetarian sources, 8) I trust that food manufacturer does not add too many unpermitted food additives to their products. While, avoidance questions were as follows: 1) Foods containing additives are harmful to human health 2) I do not mind paying more for additive-free food 3) Natural food additives are safer than artificial ones, 4) I pass certain foods up because they contain food additives, 5) The more natural the products are, the higher the quality of nutrients and vitamins, 6) I pay attention during grocery shopping to ensure they are of natural origin, 7) I gladly pay a higher price for natural foods, 8) I feel good when I eat natural foods, 9) Natural foods taste better than processed foods, 10) Natural foods are better for my health.

### 2.4. Reliability and validity testing of the food additives acceptance and avoidance questionnaire

#### 2.4.1. Acceptance of food additives.

The Kaiser-Meyer-Olkin measure indicated adequate sampling adequacy (KMO = 0.724), and Bartlett’s test supported the factorability of the correlation matrix (X2 = 924.152; df = 28; p < 0.001). Principal component analysis with Promax rotation identified two factors, which together accounted for 50.94% of the total variance. The internal consistency of the scale was acceptable, with a Cronbach’s alpha of 0.718 (Table 1).

**Table 1:** Factor analysis of food additives acceptance (Promax rotated component matrix).

Without food additives, the variety of food products would be severely limited.	1	0.528
All foods contain additives, so there is no way to avoid them	1	0.67
The additive content of foods labeled as “Diet” is very low	1	0.661
Food additives obtained from Genetically modified organisms (GMOs) can be used safely in convenience foods	1	0.695
The food label does not help identify the different sources of food additives	1	0.436
Food products labeled as “Halal Certified “assure Muslim faith that the ingredients, including additives, meet their religious requirements	1	0.874
The food label “Suitable for vegetarians” means that all ingredients and food additives are from vegetarian sources.	2	0.901
I trust that food manufacturer does not add too many unpermitted food additives to their products.	1	0.657

#### 2.4.2. *Avoidance of food additives.*

The Kaiser-Meyer-Olkin measure indicated excellent sampling adequacy (KMO = 0.871), and Bartlett’s test supported the factorability of the correlation matrix (X2 = 2099,94; df = 45; p < 0.001). The principal components analysis with Promax rotation extracted two factors explaining 53.91% of the variance. Reliability analysis yielded a Cronbach’s alpha of 0.837, indicating good internal consistency (Table 2).

**Table 2:** Factor analysis of food additives avoidance (Promax rotated component matrix).

	Highest loading component	Loading
Foods containing additives are harmful to human health	2	0.696
I do not mind paying more for additive-free food.	2	0.621
Natural food additives are safer than artificial ones.	2	0.697
I pass certain foods up because they contain food additives	2	0.611
The more natural the products are, the higher the quality of nutrients and vitamins	1	0.805
I pay attention during grocery shopping to ensure they are of natural origin	1	0.719
I gladly pay a higher price for natural foods	1	0.737
I feel good when I eat natural foods.	1	0.828
Natural foods taste better than processed foods	1	0.747
Natural foods are better for my health	1	0.831

### 2.5. Ethical approval

The study received ethical approval from the Ethics and Research Committee at the Lebanese International University (LIU) under the code 2023ERC-129-LIUSOP. The study complies with the Helsinki Declaration [43]. Before proceeding with the survey, participants provided informed consent by clicking a box, which confirmed their understanding of the study objectives and their freedom to withdraw at any time. Consent was further implied through the completion and submission of the online survey. All collected data were anonymous.

### 2.6. Statistical analysis

Data were analyzed using SPSS software (version 25). Descriptive analysis included counts and percentages for categorical variables, while means (+/-SD) were used for continuous measures. Firstly, the sample was normally distributed as checked by visual inspection of the histogram and verified by the normality line of the regression plot and scatter plot of the residual. Then, the independent-sample t-test was used to compare the means between the two groups (i.e., FAs acceptance and FAs avoidance), and the ANOVA test was applied to compare the means. All variables with a p-value < 0.05 in the bivariate analysis were included in the linear regression models to account for potential confounders. A multivariate analysis (linear regression) was carried out using the enter method, taking the acceptance and avoidance of FAs as the dependent variable. A p-value<0.05 was considered significant.

## 3. Results

### 3.1. Socio-demographic characteristics

The total number of participants enrolled was 601. The sample included 255 males (42.4%) and 346 females (57.6%). Respondents were predominantly younger than 25 years old (n = 322, 53.6%). Married participants accounted for 191 respondents (31.8%). The majority held a university degree (77.9%), and in terms of occupation, a small percentage of respondents (6.8%) were dietitians (Table 3).

**Table 3:** Sociodemographic characteristics of participants (n = 601).

Variable	N (%)
**Gender**	
Male	255 (42.4%)
Female	346 (57.6%)
**Age**	
Less than 25	322(53.6%)
Between 26–39	124(20.6%)
40 and above	155(25.8%)
**Marital status**	
Single	410 (68.2%)
Married	191 (31.8%)
**Have children**	
Yes	417 (69.4%)
No	184 (30.6%)
**University degree**	
Yes	468 (77.9%)
No	133(22.1%)
**Being a dietitian**	
Yes	41 (6.8%)
No	560 (93.2%)

### 3.2. Knowledge level, understanding of food labels, and drivers of food purchasing choices

**Fig 1:**
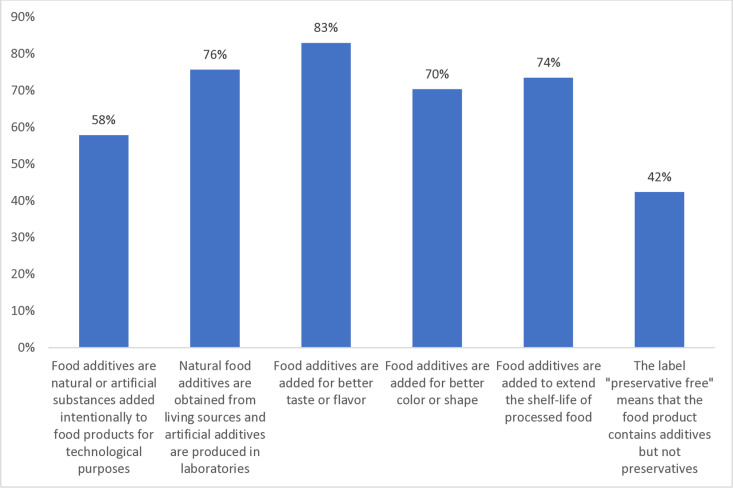
Knowledge rate of food additive definition and labeling.

The assessment of the knowledge of FAs showed varying levels of consumer understanding across different aspects. The knowledge level of the basic definition of FA was good, and consumers correctly identified its purpose (57.9%). Knowledge of the FA origin of natural and artificial FAs showed “very good” accuracy (75.7%), while the understanding of FAs’ role in enhancing taste and flavor was excellent (83%) and showed a “very good” awareness level of their use in improving color and shape (70.4%) and extending shelf-life (73.5%). However, the understating of labeling was nearly borderline (42.4%) (Fig 1).

**Fig 2:**
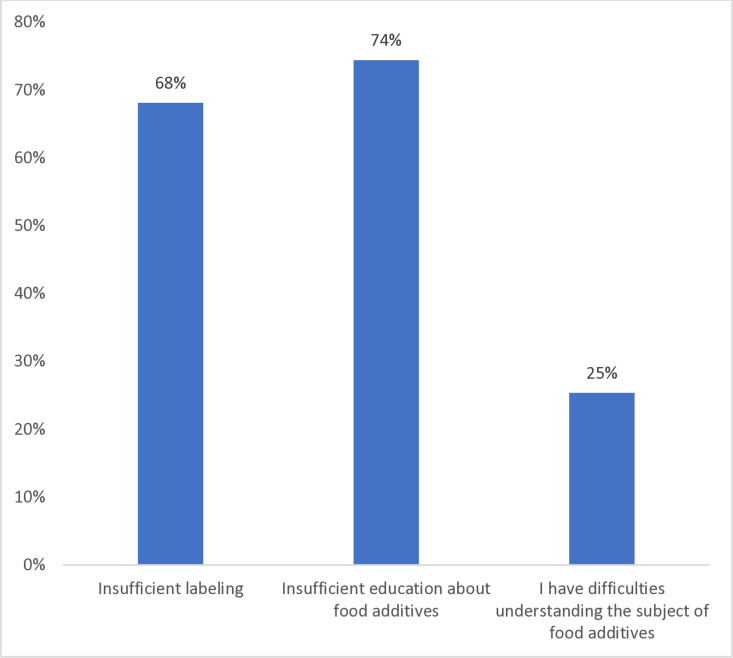
Perceived challenges in understanding food labels.

Results showed that the perceived challenges in understanding food labels were predominantly related to insufficient education about FAs (64.9%), followed by insufficient labeling (59.4%), and a limited number of participants (22.1%) indicated difficulties understanding FAs subject (Fig 2).

**Fig 3:**
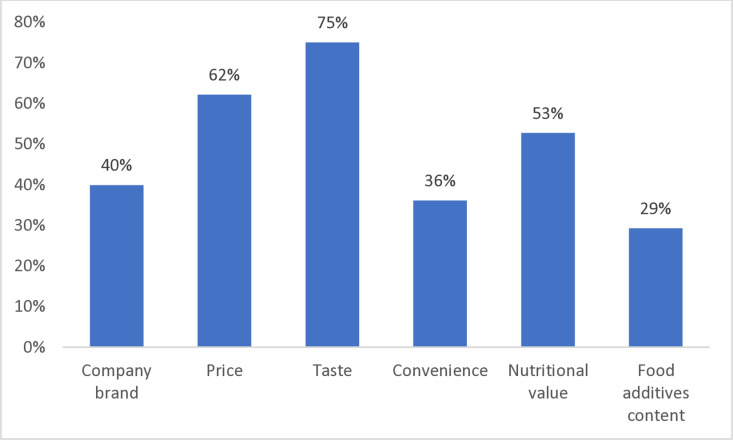
Drivers of food purchasing choices.

Data also showed that Lebanese consumers consider mainly taste (75%) and price (62.2%), while FA content has limited influence on food choices (29.3%) (Fig 3).

### 3.3. Bivariate analysis

The association between the socio-demographic characteristics and the level of knowledge about FAs indicated significant differences in factors including gender, age groups, marital status, having children, a university degree, and being a dietician (p < 0.05). Males demonstrated greater knowledge levels of FAs than females (p < 0.001) and exhibited higher levels of FA knowledge (58.6%) categorized as “excellent” and a lower level (27.4%) classified under the “poor” category. Concerning the age groups, participants less than 25 years had a higher level of knowledge (72.6%) that fell into the “poor” category than other age groups while, interestingly, showing a higher level classified under the “excellent” category (38.9%). Participants holding a university degree scored higher in the “poor” (74.2%) and “excellent” (87.9%) knowledge level categories. Similarly, single participants and those with no children also scored high in both categories. Being a dietician showed a limited “poor” knowledge level category (1.6%). However, not being a dietician scored higher in the “excellent knowledge level category (82.2%) than being a dietician (17.8%) (Table 4).

**Table 4:** Socio-demographic factors influencing knowledge levels about food additives.

Description	Level of knowledge				Significance		
	Poor	Good	Very good	Excellent	X2	df	*P* value
**Gender**							
Male	17 (27.4%)	78 (31.8%)	68 (49.6%)	92 (58.6%)	36.69	3	**<0.001**
Female	45 (72.6%)	167 (68.2%)	69 (50.4%)	65 (41.4%)			
**Age groups (years)**							
Less than 25	45 (72.6%)	143 (58.4%)	73 (53.3%)	61 (38.9%)	26.03	6	**<0.001**
26-40	10 (16.1%)	44 (18%)	29 (21.2%)	41 (26.1%)			
40 and above	7 (11.3%)	58 (23.7%)	35 (25.5%)	55 (35%)			
**University degree**							
No	16 (25.8%)	62 (25.3%)	36 (26.3%)	19 (12.1%)	12.45	3	**0.006**
Yes	46 (74.2%)	183 (74.7%)	101 (73.7%)	138 (87.9%)			
**Marital status**							
Single/ widowed/ divorced	48 (77.4%)	170 (69.4%)	98 (71.5%)	94 (59.9%)	8.31	3	**0.04**
Married	14 (22.6%)	75 (30.6%)	39 (28.5%)	63 (40.1%)			
**Have children**							
No	49 (79%)	172 (70.2%)	101 (73.7%)	95 (60.5%)	9.83	3	**0.02**
Yes	13 (21%)	73 (29.8%)	36 (26.3%)	62 (39.5%)			
**Being a dietician**							
No	61 (98.4%)	240 (98%)	130 (94.9%)	129 (82.2%)	42.04	3	**<0.001**
Yes	1(1.6%)	5 (2%)	7 (5.1%)	28 (17.8%)			

Results showed that male participants (Mean: 27.47; SD: 5.58), those between 26 and 40 years (*Mean*: 27.77; SD: 5.72), and not a dietician (*Mean*: 26.94; SD: 5.33) had significantly higher acceptance of food additives than females (*p* = 0.007), other age groups (*p* = 0.014), and being a dietician (*p* = 0.007) respectively (Table 5).

**Table 5:** Socio-demographic factors associated with acceptance and avoidance of food additives.

Description	Acceptance of FAs	*P* value	Avoidance of FAs	*P* value
	**Mean (SD)**		**Mean (SD)**	
**Gender**				
Male	27.47 (5.58)	**0.007**	47.39 (7.15)	**0.002**
Female	26.28 (5.08)		49.27 (7.24)	
**Age groups (years)**				
Less than 25	26.62 (5.13)	**0.014**	48.20 (7.51)	0.615
26-40	27.77 (5.72)		48.85 (7.58)	
40 and above	26.78 (5.33)		48.73 (6.43)	
**Martital status**				
Single/ widowed/ divorced	26.63 (5.05)	0.341	48.69 (7.33)	0.275
Married	27.11 (5.88)		48.00 (7.10)	
**Have children**				
No	26.63 (5.08)	0.317	48.74 (7.46)	0.164
Yes	27.13 (5.86)		47.85 (6.74)	
**University degree**				
No	27.29 (4.79)	0.212	46.72 (6.73)	**0.002**
Yes	26.64 (5.47)		48.97 (7.33)	
**Being a dietician**				
No	26.94 (5.33)	**0.007**	48.31 (7.16)	0.046
Yes	24.61 (4.80)		50.66 (8.20)	

Avoidance of FAs was significantly associated with female participants (*Mean*: 49.27; SD: 7.24), those that hold a university degree (*Mean*:48.97; SD: 7.33), and being a dietician (*Mean*: 50.66; SD: 8.20) compared with males, those with no university degree (*p* = 0.002) and those who are not a dietician (*p* = 0.046) respectively.

### 3.4. Multivariable analysis

The gender and age did not show significant associations with FA acceptance. However, males were more likely to avoid FAs than females (β = 1.467, p = 0.016). Similarly, participants holding a university degree showed avoidance of FAs (β = 1.809, p = 0.012). In particular, being a dietician was significantly associated with lower FA acceptance (β = −1.875, p = 0.031) (Table 6).

**Table 6:** Predictors of acceptance and avoidance of food additives: multiple linear regression analysis.

Model 1: Acceptance of FAs is the dependent variable				
	Β*	*p-v*alue**	Confidence interval	
			Lower Bound	Upper Bound
Gender (Male/Female*)	0.898	0.059	−1.830	0.033
Age (between 26- and 40-years/ less than 25 years*)	−0.832	0.142	−1.943	0.279
Age (between 40 years and above/ less than 25 years*)	0.602	0.282	−0.496	1.699
Being a Dietician (No/Yes*)	−1.875	**0.031**	−3.574	−0.175
**Model 2: Avoidance of FAs is the dependent variable**				
	Β*	*p-v*alue**	**Confidence interval**	
			**Lower Bound**	**Upper Bound**
Gender (Male/Female*)	1.467	**0.016**	0273	2.661
University degree (No/Yes*)	1.809	**0.012**	0.396	3.223
Being a Dietician (No/Yes*)	1.572	0.182	−0.740	3.883

* β*: s*tandardized coefficient; **p-value< 0.05 is significant.

## 4. Discussion

This study revealed a complex interplay between cognitive and behavioral factors in FAs related decision making among Lebanese consumers. Our findings showed that despite high theoretical FAs knowledge (83% accuracy), consumers demonstrated poor label understanding and relied on heuristic purchasing behavior, prioritizing taste (75%) and price (62.2%) over FA content (29%) and label information analysis. Three key insights emerge from these paradoxical findings: (1) a clear disconnect between knowledge and behavior, particularly under crisis conditions, (2) socio-demographic patterns that challenge conventional assumptions, and (3) defined opportunities for targeted public health policy interventions.

### 4.1. Drivers of food choices and knowledge of food additives

The observed heuristic purchasing behavior primarily reflects Lebanon’s severe economic collapse since 2019 [43], characterized by hyperinflation and rising food insecurity. This crisis context has fundamentally reshaped consumer priorities, making affordability and accessibility the dominant factors in food selection, even among nutritionally-literate individuals.

Our analysis identified two key explanatory factors underlying the disconnect between high theoretical knowledge and suboptimal food purchasing behavior. First, economic determinants play a critical role: the ongoing crisis in Lebanon has compelled consumers to prioritize immediate survival needs, leading to a reliance on affordable processed foods regardless of their adulteration content. This economic pressure contributes to “decision fatigue,” where individuals default to simple heuristics such as price and taste rather than engaging in complex nutritional or safety assessments. Second, significant barriers to label comprehension were observed. Although participants demonstrated high theoretical knowledge about food adulteration (83%), their practical understanding of food labels was markedly lower (42.4%), diverging from findings in previous Lebanese studies. This gap can be attributed to overly technical label designs that surpass average literacy levels, the influence of misleading marketing claims (e.g., interpreting “no preservatives” as a blanket indicator of health), and behavioral exhaustion resulting from prolonged exposure to crisis conditions.

These findings align with global research demonstrating how ineffective labeling perpetuates consumer confusion while simultaneously influencing industry practices [44–46]. The Lebanese context presents an acute case of this dynamic, exacerbated by unique socioeconomic stressors.

### 4.2. Socio-demographic factors influencing FAs acceptance and avoidance

The study showed variable dynamics in the socio-demographic factors influencing FAs acceptance and avoidance. Gender and education level affected FAs avoidance, while an educational background in nutrition influenced acceptance. Notably, no association was found between FAs acceptance/ avoidance and different age groups, marital status, or having children.

#### 4.2.1. Gender disparities.

Contrary to numerous studies [36,47–49], results showed that males tended to avoid FAs use and favored natural or additive-free food products more than females. The unexpected finding necessitates further investigation to inform tailored awareness campaigns. In this study, the avoidance is influenced by a combination of factors, including risk perception, preference for natural food and willingness to pay for FAs free food.

Exploring the underlying cognitive processes that shape consumers behavior through theoretical frameworks, can provide a better understanding of the gender disparities, for example: Slovic’s Risk Perception Attitude (RPA) theory [50], can examine how individuals perceive and evaluate risks associated with FAs. This theory suggest that avoidance may be influenced by psychological factors such as dread risk (e.g., the level of fear associated with the use of FAs), unknown risks (e.g., the perceived lack of familiarity or understanding of FAs), and the perceived exposure, rather than only by objective scientific assessment of their safety [50]. In other terms, males might exhibit stronger avoidance behavior toward FAs, potentially considering them more dreadful or less understood risks than familiar food components. In addition, an integrated model of the theory of planned behavior and the value-belief-norm (VBN) theory can help evaluate consumers trust in natural food [51].

By integrating these theories and focusing on gender differences, future research can provide an explanation of the cognitive processes underlying FA avoidance and predict future behaviors to inform policy-makers, food manufacturers and health educators in providing targeted public health communication strategies.

#### 4.2.2. Age and education.

Concerning age, individual less than 25 years showed a higher “poor” knowledge score than other age groups, similar to studies conducted in the UAE [13] and previously in Lebanon [41]. While the more than 25 years age groups exhibited a wide distribution across knowledge levels however, these results should be interpreted with caution due to the smaller sample size of these groups compared to the less than 25 years age group. Nevertheless, these findings underscore the need to address educational gaps early on, emphasizing the importance of integrating food literacy into school curricula to better equip younger generations in making informed and health-conscious food choices.

With regards to education, having a higher education, particularly being a dietician influenced FA avoidance and lower acceptance. However, the relationship between the level of education and FA knowledge, perception and attitude toward FAs yielded variable results in the literature [13,24,32,36].

In this study, educated people scored high in the different levels of knowledge from “poor” to “excellent”. This means that the educational background (i.e., Health or non-health education), may impact the level of knowledge, and attitude toward FAs. For example, being a dietician did not influence avoidance of FAs, but it was associated with lower acceptance than not being a dietician.

Multiple misconceptions exist about FAs mainly due to risk perception of their short-term and may be long-term impact on health [13,24], or because research on FAs safety is based predominantly based on animals testing [22], in addition to the influence of social media and infodemics [52,53]. Although processed food and ultra-processed food containing FAs were associated with a high risk of cardiometabolic and other non-communicable diseases [1–5], these products did not only contain FAs. The way to address the knowledge gap is effective communication, awareness campaigns, consumers education that must start at a younger age, and enhancing trust in regulators [54,55].

### 4.3. Implications to public health policy

The findings indicate four pathways for public health intervention: First, regulatory measures should mandate standardized, visually intuitive, user-friendly labeling formats or government-certified mobile apps to decode additives. Second, capacity-building initiatives tailored to different educational levels and age groups could incorporate FA literacy modules into school curricula, nutrition programs, community workshops, and awareness campaigns. Third, dietitians can serve as community educators to effectively shift consumption patterns, while media campaigns should address the taste-price-FA content identified in consumer decision-making. Fourth, a multidisciplinary task force is needed to address misinformation on social media that exacerbates label misunderstanding.

#### 4.3.1. Study limitations.

Several limitations must be considered when interpreting these findings. First, the use of snowball sampling likely introduced selection bias, potentially limiting the representativeness of the sample. Second, reliance on self-reported data raises concerns about social desirability bias, recall bias, and inaccuracies in reporting socio-demographic characteristics or past behaviors. Third, the study’s exclusive focus on Lebanese consumers during a period of severe economic and political instability, which may restrict the generalizability of results to other cultural or socioeconomic contexts. The analysis did not account for confounding variables such as income level, food insecurity, or cultural dietary norms, which may significantly influence attitudes toward food additives. The small subgroup sample sizes (i.e.,., only 41 dieticians) and uneven age distribution further limit the reliability of some comparisons. Methodologically, administering the questionnaire exclusively in English may have excluded broader populations, particularly older or less educated individuals. Finally, the quantitative design necessitates deeper exploration of motivations; future mixed-methods research incorporating qualitative interviews could provide deeper insights into consumer decision-making processes.

## 5. Conclusion

The study provides valuable insights into the knowledge level and attitudes of Lebanese consumers toward FAs. Our findings highlighted an important gap in understanding FAs based on socio-demographic factors. To promote informed choices among Lebanese consumers, the engagement of the government officials, academic sectors, media representative, school educators, healthy policy makers is crucial. Enhancing advocacy, accountability and communication between these stakeholders and also international organizations and regulatory bodies, can secure food safety and protect consumers. The study highlighted the pressing need for food labeling policies, fostering multidisciplinary collaboration, managing infodemics and misconceptions, encouraging research and designing targeted of education and awareness campaigns. The ultimate goal is an informed consumer and improved public health outcomes despite the current barriers and challenges faced in Lebanon.

## Supporting information

S1 File Inclusivity-in-global-research-questionnaire. (DOCX)
