# Correction: Socio-demographic influence of food additives acceptance and avoidance among the Lebanese population: A cross-sectional study

**DOI:** 10.1371/journal.pone.0336265

**Published:** 2025-11-04

**Authors:** Reham E. Kotb, Souheil Hallit, Farah Asmar, Melissa Fakhry, Chadia Haddad, Pascale Salameh, Samar Younes, Mohammed Bahlol, Mohamad Abdelkhalik, Michelle Cherfane, Katia Iskandar

The eighth author’s last name is incorrect. The correct name is: Mohammed Bahlol.
